# A Novel Acoustic Sensor Approach to Classify Seeds Based on Sound Absorption Spectra

**DOI:** 10.3390/s101110027

**Published:** 2010-11-09

**Authors:** Vicent Gasso-Tortajada, Alastair J. Ward, Hasib Mansur, Torben Brøchner, Claus G. Sørensen, Ole Green

**Affiliations:** Department of Biosystems Engineering, Faculty of Agricultural Sciences, Aarhus University, Blichers Allé 20, P. O. Box 50, DK-8830 Tjele, Denmark; E-Mails: vicent.gassotortajada@agrsci.dk (V.G.T.); alastair.ward@agrsci.dk (A.J.W.); hasib.mansur@agrsci.dk (H.M.); torben.brochner@agrsci.dk (T.B.); claus.soerensen@agrsci.dk (C.G.S.)

**Keywords:** seed, acoustic, sound, absorption, non-destructive, classification, identification, multivariate statistics

## Abstract

A non-destructive and novel *in situ* acoustic sensor approach based on the sound absorption spectra was developed for identifying and classifying different seed types. The absorption coefficient spectra were determined by using the impedance tube measurement method. Subsequently, a multivariate statistical analysis, *i.e*., principal component analysis (PCA), was performed as a way to generate a classification of the seeds based on the soft independent modelling of class analogy (SIMCA) method. The results show that the sound absorption coefficient spectra of different seed types present characteristic patterns which are highly dependent on seed size and shape. In general, seed particle size and sphericity were inversely related with the absorption coefficient. PCA presented reliable grouping capabilities within the diverse seed types, since the 95% of the total spectral variance was described by the first two principal components. Furthermore, the SIMCA classification model based on the absorption spectra achieved optimal results as 100% of the evaluation samples were correctly classified. This study contains the initial structuring of an innovative method that will present new possibilities in agriculture and industry for classifying and determining physical properties of seeds and other materials.

## 1. Introduction

### 1.1. Background

Seed classification and variety recognition are important operations in the food production and processing industries. For instance, in a seed handling facility, seeds are graded, cleaned and identified for proper binning and shipping according to buyer’s specifications [1]. Seeds are traditionally manually identified for binning, but this practice is tedious, labour-consuming and imprecise [2]. More accurate methods, such as polyacrylamide gel electrophoresis, high performance liquid chromatography, protein electrophoretic and molecular marker, have been used for seed varietal identification [3–5] although they are destructive, time consuming and relatively costly techniques [6]. To facilitate the automation of the process, rapid, *in situ* and non-destructive identification of seed type and variety is required as a way to unload and direct automatically the seeds to the correct receiving bin within the seed handling facility.

Some potential prospective methods for automating the seed identification process already exist. These include near-infrared (NIR) spectroscopy, a non-destructive and fast developing technique that has been applied to identify the chemical composition of seeds [7–9]. However, few studies have focus on seed type or variety identification [10]. On the other hand, digital image processing techniques offer a new option in order to obtain quick classification results [1,3,11–14]. Nevertheless, the natural variability in seed appearance (regarding shape, colour and texture), as well as the existence of dust particles and uneven backgrounds still make the recognition of seeds a challenge for any machine vision system [3,15]. Therefore, alternative non-destructive and *in situ* techniques to identify and classify seed types and varieties need to be developed.

The recent development of acoustic and digital analysis techniques offers innovative tools for an emergent number of industrial applications, especially in the food industry [16]. Ultrasound waves are widely used for the quality assessment of fruits, drinks and oils [16,18–21]. However, sound waves have considerable advantages over ultrasound in terms of being able to assess large materials widths and being relatively inexpensive [17]. Sound waves are used to characterize the texture of nectarines, plums, and tomatoes by estimating the sound velocity through these materials [22,23]. Green *et al.* [24] have related the sound insulation characteristics with the physical properties of different forage types. Moreover, some studies have applied acoustic methods for assessing seeds. For instance, the analysis of the acoustic signal generated by the dynamic impact of seeds has been used to determine the seed moisture content [25,26] and to detect defects and seed damages [27–29].

### 1.2. Acoustic Phenomena

As a granular porous material, heaped seeds are a highly absorbing acoustic media [30,31]. The acoustic energy transmission in a porous media can be divided into structure-borne and air-borne transmission [32]. Hikling *et al.* [31,33] found that in heaped seeds, sound waves are principally air-borne transmitted, *i.e.*, transmitted through the gas phase between seeds, since the transmission of sound through the solid seed matrix, *i.e*., structure-borne transmission, appears to be highly attenuated and almost nonexistent, presumably due to friction between seeds.

When the air-borne sound wave comes into contact with heaped seeds, part of its energy is reflected, another fraction is transmitted through the medium and the rest is dissipated in form of heat due to thermo-viscous effects between seed particles (*i.e.*, absorption phenomenon) [34].

The acoustic behaviour of a porous material is principally governed by the physical properties of the medium. Theoretical studies have shown that the main parameters that influence the acoustic behaviour of porous media comprise porosity, air flow resistivity, tortuosity, viscous characteristic length and thermal characteristic length [32]. The latter two parameters describe the viscosity effects and the thermal changes between the solid and the fluid phases, respectively. Additional investigations have shown that at high frequencies, parameters such as porosity, tortuosity and viscous and thermal characteristic lengths play a key role, while at low frequencies, porosity, air flow resistivity and thermal permeability defined by Lafarge *et al.* [35] are the key parameters [32]. Experimental studies have proven that the acoustic behaviour of porous granular media can be basically described in terms of porosity, air-flow resistivity and tortuosity [36,37]. Furthermore, Guo *et al.* [30] found that in heaped seeds, the mechanism of sound absorption significantly depends on particle size and shape, which are parameters related with the previous ones.

### 1.3. Aim of the Study

The aim of this study is to develop a novel non-destructive and *in situ* sensor approach based on sound absorption phenomena for identifying and classifying different seed types by means of multivariate statistical analysis. The proposed method is based on the already shown positive potentials of using multivariate statistical classification methods for analysing acoustical spectra of different agricultural materials [24].

## 2. Materials and Methods

The experimental assessment was based on the calculation of the absorption coefficient (α) (ratio of absorbed sound energy to the incident sound energy) spectrum of different types of seeds and its respective multivariate statistical analysis for developing a seed classification model.

### 2.1. Absorption Measurement Principle

**Figure 1.**
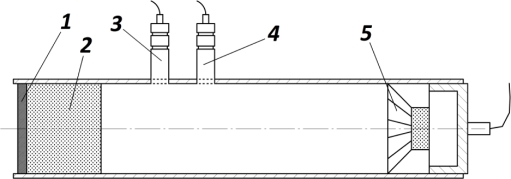
Impedance tube. Where 1 is a rigid termination, 2 the test sample, 3 and 4 the microphones and 5 the loudspeaker.

The absorption coefficient (α) was determined by means of the impedance tube method. The impedance tube consists of a hollow metallic cylinder where the test sample is placed at one end of the tube, while at the other end plane sound waves are generated by a loudspeaker (see Figure 1).

The sound pressure is measured by two microphones at two different locations near to the sample. Then, the calculation of α is performed by invoking a transfer function based on the pressure differences between the two microphone positions [38]. These pressure differences depend on the degree of reflection and absorption of the standing waves that are influenced by the sample under test.

### 2.2. Equipment Specifications

The impedance tube set used in this study (SCS9020 Kundt tube, S.C.S. Controlli e Sistemi, Italy) was manufactured according to the ISO 10534-2 and ASTM E1050-98 standards. This set consisted of two impedance tubes with different internal diameters (28 and 100 mm) and different microphone locations. The two different tubes were developed for working at two different sound frequency ranges in order to ensure the creation of plane waves and subsequently the execution of an accurate phase detection by the microphones. The large diameter tube is used for working at the low frequency range (LF) which presents an upper limit of around 1,000 Hz, while the small diameter tube is used at the higher frequency range (HF).

Two cylindrical sample-holders for holding the seeds inside each respective tube were manufactured. Polymethyl methacrylate (PMMA) was used for this purpose. Both sample-holders had a length of 200 mm that corresponds to the analysed sample thickness. The sample-holder of the LF tube had an external diameter of 100 mm and 2 mm of thickness, and the HF one had an external diameter of 27.5 mm and 3 mm of thickness. The apertures of each sample-holder were sealed by using metal wire mesh. The wire had a diameter of 0.1 mm and a distance between wires of 0.15 mm. In order to facilitate the insertion of the seeds, a lateral orifice was performed at 40 mm from one of the edges with a 48 and 9 mm of diameter for the LF and the HF sample-holders respectively. For minimizing any acoustic influence of this orifice, it was ensured that the sample-holder edge adjacent to the orifice was placed opposite to the sound source. A 0.5 mm air-gap was present between the sample-holder of the HF and the impedance tube. The LF set did not present any significant air-gap.

The noise generator unit (MESA G10, S.C.S. Controlli e Sistemi, Italy) was responsible for generating a white noise signal which was subsequently amplified by a power amplifier (MESA V31, S.C.S. Controlli e Sistemi, Italy) and sent to the loudspeaker integrated in the respective impedance tube. The microphones used were 1/4 inch pressure type (40 BP, G.R.A.S. Sound & Vibration, Denmark). The sensitivity and the phase mismatch were calibrated at an earlier stage. The acoustic signal monitored by the microphones was subsequently conditioned by a signal acquisition unit (Symphonie, 01 dB-Stell, France). The system had a 16 bits resolution with a maximum sampling frequency of 51.2 KHz. For data processing, SCS80FA software (S.C.S. Controlli e Sistemi, Italy) in conjunction with dBFA suite software (01dB-Metravib, France) was used.

The α spectra were calculated following the ISO 10534-2 and ASTM E1050-98 standards. The spectra were determined independently for the low frequency range (50–997 Hz) and the high frequency range (800–4,996 Hz) with a precision of 3.13 Hz. Additionally, both spectra were linked by the SCS80FA software in a complete spectrum (50–4,250 Hz) by using a smoothing function within the overlapping frequencies.

### 2.3. Sample Preparation

Seven different types of seeds were used in the experiment. They consisted of oat (*Avena sativa*), barley (*Hordeum vulgare*), maize (*Zea mays*), wheat (*Triticum*), sunflower (*Helianthus annuus*), soya bean (*Glycine max*) and canola (*Brassica napus*). In total, 70 samples (10 samples per seed type) were investigated. The samples of each seed type were numbered randomly from 01 to 10. Note that samples within the same seed type were taken from the same seed lot. The seed mean diameter of each sample was determined by using ASTM sieve series with apertures of 5.6, 4.0, 2.8, 2.0, 1.4 and 1.0 mm. Additionally, a qualitative description of the seed particles sphericity was determined based on visual inspection. During the sample preparation, each sample bag was sealed to ensure a constant moisture content during the course of the experiment. When filling the sample-holders with the respective seeds, any significant empty space inside the sample-holder was avoided. Next, the density of each sample was determined.

### 2.4. Statistical Analysis

The SIMCA (Soft Independent Modelling by Class Analogy) statistical classification method was used to classify the different seed samples. All analyses were carried out using The Unscrambler v9.8 software (CAMO Software A/S, Norway).

All the samples were initially analysed by principal component analysis (PCA). This was to make a preliminary investigation of any similarities or grouping within the same seed type and/or between different seed types. PCA is the process of decomposition of the spectral data into structural and noise parts. The largest variance in the spectral data was labelled the first principal component (PC1), and the next largest variation was labelled the second principal component (PC2). PCs of higher order will progressively describe more of the noise and less of the structure of the samples data. Once the PCA model was generated, it was validated using the leave-one-out cross validation method.

The training data set used for generating the SIMCA classification model comprised 8 of the 10 samples of each seed type (samples numbered from 03 to 10). This data was recorded as a matrix of columns representing α values at the respective frequencies and rows representing the different samples. Then, the training data matrix for each seed type was analysed using PCA, producing separate PCA models for each seed type. These PCA models were again validated using the leave-one-out cross validation method.

In order to evaluate the identification ability of the SIMCA classification method, the samples 01 and 02 of each seed type (evaluation samples) which were not included in the training data set, were analysed and classified in a particular seed type based on their similarity with respect to the particular PCA class model.

Two different classification models were compiled, one based on the full spectral data (50–4,250 Hz) and another based on a reduced spectral data (100–997 Hz) which, as will be explained in Section 3.2, only includes the frequency range with the lowest standard deviation of α.

## 3. Results and Discussion

### 3.1. Spectral Patterns Analysis

**Figure 2.**
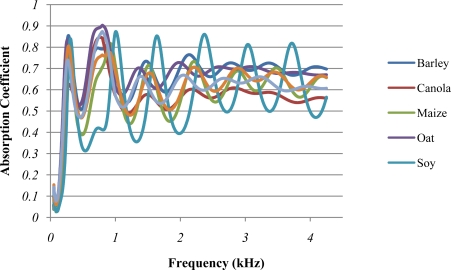
Absorption coefficient average spectra of the different seed types.

**Figure 3.**
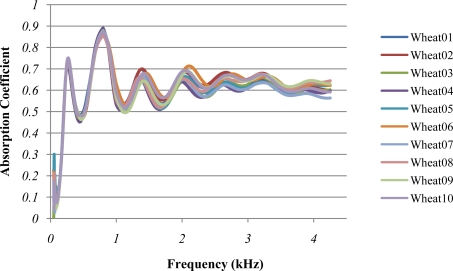
Absorption coefficient spectra of the wheat samples.

Figure 2 presents the absorption coefficient (α) average spectra of different seed types which have been analysed. These spectra follow a succession of several absorption peaks with very characteristic patterns in terms of α average value, resonance frequencies and amplitude of the resonance oscillations. These patterns diverge significantly when they are compared among different seed types, but they are very similar within samples of the same seed type (see Figures 2 and 3). In contrast, it is found that at the lowest frequencies (from 50 to 100 Hz), large divergences exist even between samples of the same seed type.

Table 1 shows the α average value for each specific seed type, which was determined by calculating the α average of the different samples of each seed type at all frequencies within the respective range. At the low frequency range (LF) and in the case of comparing the α average of seed types with approximately similar shape, for instance soya bean-canola, maize-wheat or sunflower-barley (see Table 1), the types with smaller particle size presented a larger sound absorption compared to the bigger ones. Additionally, if the α average of seed types with approximately similar size is compared, for instance oat-barley or sunflower-maize-soya bean, non spherical types showed a larger absorption than the spherical ones. These results are in accordance with Guo *et al.* [30]. The present findings confirm that the sound absorption phenomenon is principally governed by the size and the shape of the particles, which are parameters that define samples porosity, tortuosity and air flow resistivity. It is pertinent to note that the inverse relation found between particle size and α may be limited to the particle size range analysed in this study. This hypothesis is based on results from acoustic investigations of other granular materials. For instance, Swenson *et al.* [39] found a similar behaviour in granular materials with similar and larger particle sizes, *i.e*., gravels (2–25 mm). Nevertheless, researches on granular materials with smaller particle sizes, *i.e*., sands (0.25–1.60 mm) [40] or synthetic rubbers and minerals (0.40–3.50 mm) [34], found the opposite phenomenon, which was an increase of the sound absorption when increasing the particle size.

At the high frequency range (HF), in general, the size and shape phenomena were still observable with the notion that they were less clear than at the low frequency range. As mentioned earlier, this finding may be explained by changes in the influence of some physical parameters that depend on the frequency range under analysis [32] or probably by the existence of significant errors at the HF as will be discussed afterwards.

In the case of comparing the density variations among samples with the same seed type, and therefore with similar shape and size, no clear trend was found between α and density (data not shown). Nevertheless, studies analysing granular materials with a smaller particle size found an inverse relationship between absorption and density [34,40], which suggests that this phenomenon may also be dependent on the particle size range analysed.

When the resonance frequencies of the seed type average spectra were analysed (see Figure 2), it was found that the resonance peak frequency of analogous order between seed types with approximately similar shape (as an example, the frequency of the 3^rd^ resonance peak of maize and wheat) was lower for the smaller seed types. Whereas, when comparing seeds with approximately similar size the more the type was non-spherical, the lower was the resonance peak frequency of analogous order. Besides, when comparing the same seed type combinations as before, smaller and non-spherical types presented a lower amplitude of the resonance oscillations.

It is relevant to mention that Guo *et al.* [30] observed that the number of resonance peaks increases with the sample thickness. This finding is in agreement with the results of the present study since wheat spectrum showed seven peaks for a sample thickness of 20 cm (see Figure 2), while Guo *et al.* [30] found, from 100 to 5,000 Hz, 1, 2 and 3 resonance peaks for wheat sample thicknesses of 2, 4 and 8 cm, respectively.

The average of the α standard deviation (SD) presented in Table 1 was determined firstly by calculating the α SD of the different samples at each specific frequency and then by averaging the SD values of the different frequencies analysed. This data showed that the α SD of the diverse seed types was significantly different. When comparing the α SD of seed types with approximately similar shape (soya bean-canola, maize-wheat or sunflower-barley), α SD presented a direct relationship with particle size. This finding may be explained in terms of the reduced number of seeds that can be introduced in the sample-holder when dealing with big seed particles, which results in an increased influence from the seed heterogeneities. In contrast, comparing seeds with similar size (e.g., oat-barley-wheat or sunflower-soya bean) spherical shape seeds presented a lower SD of α than the non-spherical ones. It is assumed that seed types with non-spherical shape will present increased possibilities of seeds arrangement and therefore exhibit more absorption variability than the samples with spherical shape. The phenomenon of seeds arrangement has a very important function in terms of sound absorption, firstly due to the influence on tortuosity and therefore on the sound transmission path, and secondly due to the variability of number of seeds that can be introduced per sample and subsequently its effect on sample density. This hypothesis is confirmed when analysing the SD of the samples density (see Table 1), where the more spherical the seed particle, the lower the SD of the samples density. It is relevant to mention that the observed clogging problems of the sample holder mesh due to the presence of seed dust could also have contributed to a general increase of the α SD. Besides, the high variation found in maize may also be explained by the existence of foreign bodies and fragmented seeds in these samples.

Large differences in the SD of α were also found between both frequency ranges (see Table 1). In general, the α SD at HF was approximately double that at LF. This occurrence could be caused either by changes in the influence of some physical parameters when the wavelength becomes comparable to the pore size, by the reduced number of seeds that can be placed inside the small diameter sample-holder and therefore by the increase of sensitivity to seed heterogeneities and arrangement, or merely by errors enhanced by unsuitable sample-holder features observed only in the HF sample-holder. One of these unsuitable features was the existence of an air-gap between the HF tube and its respective sample-holder that could influence significantly the spectral results, as previous studies using the impedance tube method have shown [41].

### 3.2. Multivariate Statistical Analysis

The SIMCA classification model based on the reduced spectral data (100–997 Hz) produced optimal results for classifying the different evaluation samples. This spectral data only includes the frequency range with the lowest α standard deviation which, as seen previously, is the LF (50–997 Hz) minus the lowest frequency extreme (50–100 Hz). In contrast, the performance of the classification model based on the full spectral data (50–4,250 Hz) was poorer since some samples of barley and sunflower were wrongly classified. This occurrence may be explained by the relative similarities between barley and sunflower spectra (see Figure 2) and the high SD found at the HF, probably enhanced by the sample-holder air gap. Table 2 shows the performance of the classification model based on the reduced spectral data where all the evaluation samples (rows) were allocated only for the correct PCA model membership (columns).

**Figure 4.**
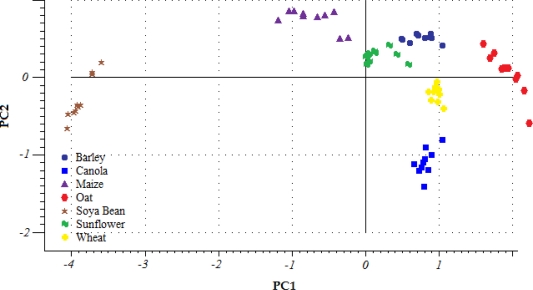
PCA score plot of the different seed samples with respect to PC1 and PC2.

The classification model results were in accordance with the initial PCA of all samples, the score plot of which is shown in Figure 4. This shows a significant grouping of the diverse seed types with respect to PC1 (x axis) and PC2 (y axis). 95% of the total spectral variance was accounted for by the first 2 principal components, where 85% was described by PC1 and 10% by PC2. Most seed types were clearly defined with respect to PC1, especially soya bean. On the other hand, barley, wheat and canola overlapped along the PC1 axis, but they were clearly differentiated with respect to PC2. Score points directly opposite each other along a particular axis are inversely correlated. For instance, soya bean and oat samples were inversely correlated with respect to PC1, which is a fact that is in agreement with the large differences found between the spectral patterns of both seed types (see Figure 2). In contrast, sunflower and barley showed the smallest PCA model distance due to the relative similarity of their spectral patterns.

## 4. Conclusions

The principal findings derived from this study included:

- The sound absorption coefficient (α) spectra of different types of seeds presented characteristic and recognisable patterns in terms of α average value, resonance frequencies, and amplitude of the resonance oscillations.
- Seed size and shape were the main governing parameters regarding the sound absorption mechanism in seeds. As a general rule, seed particle size and sphericity were inversely related with α. In addition, an influence of these parameters on the resonance frequencies and on the resonance oscillations amplitude was also found.
- The inverse relationship existing between particle size and sound absorption was limited by the particle size range under analysis, since in granular materials with smaller particle size a direct relationship was found.
- The seed arrangement variability, which was found to be related with seed sphericity, has an important function in terms of sound absorption due to its influence on sample density and tortuosity.
- Principal component analysis (PCA) presents reliable grouping capabilities within the diverse seed types. Furthermore, the SIMCA classification model based on the α spectra was able to achieve optimal classification results of the different seed samples.

In order to improve the proposed method, special attention must be paid to the construction of the sample-holders, specifically by preventing air-gaps and by increasing the mesh aperture size to avoid clogging problems and acoustic interferences. Additionally, new methodologies must be applied while filling the sample-holder as a way to reduce the seed arrangement variability, for example, by means of vibrational techniques. Further work must focus on developing multivariate statistical models based on acoustic spectra to determine seed physical properties, for instance seed particle size, weight and water content.

The current study is the initial structuring of an innovative method development that will present new possibilities in agriculture and industry for classifying and determining physical properties of seeds and other materials by means of inexpensive, non-destructive and *in situ* measurements methods.

## Figures and Tables

**Table 1.** Absorption coefficient average and standard deviation (SD), particle size, sphericity and samples density of the different seed types at the low frequency range (LF) and the high frequency range (HF). Note that the higher number of asterisks, the more spherical is the particle.

	***ABSORPTION COEFFICIENT***				***PARTICLESIZE****(mm)*		***SHAPE***	***DENSITY****(kg/m3)*			
	***LF***		***HF***					***LF***		***HF***	
	*Average*	*SD*	*Average*	*SD*	*Average*	*SD*	*Sphericity*	*Average*	*SD*	*Average*	*SD*
***Barley***	0.630	0.015	0.689	0.028	2.926	0.010	**	606.0	5.22	606.6	10.32

***Canola***	0.597	0.014	0.565	0.033	1.951	0.002	****	633.7	7.69	623.4	8.45

***Maize***	0.542	0.027	0.619	0.060	5.326	0.037	***	698.2	15.74	664.1	15.32

***Oat***	0.671	0.024	0.680	0.025	2.647	0.007	*	564.4	10.63	583.5	10.48

***Soya bean***	0.403	0.017	0.615	0.047	5.599	0.001	****	792.6	8.93	735.4	5.19

***Sunflower***	0.590	0.020	0.631	0.048	4.850	0.082	**	410.7	4.29	422.7	6.55

***Wheat***	0.608	0.011	0.619	0.022	3.112	0.005	***	712.8	4.65	695.2	3.96

**Table 2.** Classification table of the evaluation samples (samples of each seed type numbered 01 and 02). The table is marked to show in which of the PCA models (columns) the evaluation samples (rows) were best fitted.

	***Barley model***	***Canola model***	***Maize model***	***Oat model***	***Soya Bean model***	***Sunflower model***	***Wheat model***
*Barley01*	*						
*Barley02*	*						
*Canola01*		*					
*Canola02*		*					
*Maize01*			*				
*Maize02*			*				
*Oat01*				*			
*Oat02*				*			
*SoyaBean01*					*		
*SoyaBean02*					*		
*Sunflower01*						*	
*Sunflower02*						*	
*Wheat01*							*
*Wheat02*							*
